# Savine in the Treatment of Habitual Abortion

**Published:** 1851-03

**Authors:** 


*Savine in the Treatment of Habitual Abortion.* By Dr. Metsch.—When the disposition to abortion is dependent upon a diminished vitality of the uterine system, or functional weakness of its nutritive vessels, Dr. Metsch says that medicines of a stimulant and strengthening description, acting powerfully upon the circulation of the organ, are indicated, and of all such substances *savine* is most to be relied on for this end. Of course so powerful a drug requires skilful selection of appropriate cases for its employment, or it may give rise to hyperæmia of the pelvic abdominal organs, inducing hæmorrhage, inflammation, abortion, or death itself. Local or general plethora, or serious diseases of any part, contraindicate its use. An infusion is made by adding from two to four drachms to six ounces of boiling water, a spoonful being given morning and afternoon during the intervals between the menstrual periods. On some occasions, before commencing with it, catarrhal or gastric disturbances have to be allayed, as also irritation dependent upon congestion, rheumatism, or disorder of the nervous system. Small general or local bleedings, emetics, aperients, tepid baths, or friction of the surface, are required in different cases. So, too, regulation of diet, abstinence from sexual excitement, rest in the horizontal position as long as pain is present, are then indicated.

If the disposition to abortion depends upon an augmented irritability, and contractibilty—a condition not always opposed to the first named—the savine does not alone suffice, but a medicine is required that exerts a special effect in regulating uterine irritability, the ergot of rye, which should be added to the infusion in the proportion of one to two, when former miscarriages have been induced by the primary contraction of the womb without preliminary hæmorrhage. Another modification in the prophylaxis is to be made when former abortions have been attended with great urinary irritation, in which case six drops of Tr. Lyttæ should be added to each dose. When, prior to former abortions, there has been great disturbance of the digestive organs, very small doses of ipecacuanha may be alternated with the above.

The savine is also found useful in various chronic diseases of the female genital organs, connected with vascular and secretory torpor, especially in passive hæmorrhages and leuchorrhœa. In the same way it is of good service, conjoined with mechanical means, in prolapsus uteri consequent upon frequent or difficult labors and abortions. — *Zeitochrift für Gerburtkunde, Band xxxvi;* and *Brit. and For. Med. Rev.*

